# From Genomic Exploration to Personalized Treatment: Next-Generation Sequencing in Oncology

**DOI:** 10.3390/cimb46110744

**Published:** 2024-11-06

**Authors:** Vishakha Vashisht, Ashutosh Vashisht, Ashis K. Mondal, Jana Woodall, Ravindra Kolhe

**Affiliations:** Department of Pathology, Medical College of Georgia, Augusta University, Augusta, GA 30912, USA; vvashisht@augusta.edu (V.V.); avashisht@augusta.edu (A.V.); amondal@augusta.edu (A.K.M.); jawoodall@augusta.edu (J.W.)

**Keywords:** next-generation sequencing, whole-genome sequencing, whole-exome sequencing, RNA sequencing, personalized treatment

## Abstract

Next-generation sequencing (NGS) has revolutionized personalized oncology care by providing exceptional insights into the complex genomic landscape. NGS offers comprehensive cancer profiling, which enables clinicians and researchers to better understand the molecular basis of cancer and to tailor treatment strategies accordingly. Targeted therapies based on genomic alterations identified through NGS have shown promise in improving patient outcomes across various cancer types, circumventing resistance mechanisms and enhancing treatment efficacy. Moreover, NGS facilitates the identification of predictive biomarkers and prognostic indicators, aiding in patient stratification and personalized treatment approaches. By uncovering driver mutations and actionable alterations, NGS empowers clinicians to make informed decisions regarding treatment selection and patient management. However, the full potential of NGS in personalized oncology can only be realized through bioinformatics analyses. Bioinformatics plays a crucial role in processing raw sequencing data, identifying clinically relevant variants, and interpreting complex genomic landscapes. This comprehensive review investigates the diverse NGS techniques, including whole-genome sequencing (WGS), whole-exome sequencing (WES), and single-cell RNA sequencing (sc-RNA-Seq), elucidating their roles in understanding the complex genomic/transcriptomic landscape of cancer. Furthermore, the review explores the integration of NGS data with bioinformatics tools to facilitate personalized oncology approaches, from understanding tumor heterogeneity to identifying driver mutations and predicting therapeutic responses. Challenges and future directions in NGS-based cancer research are also discussed, underscoring the transformative impact of these technologies on cancer diagnosis, management, and treatment strategies.

## 1. Introduction

Cancer, a heterogeneous disease stemming from the accumulation of numerous genetic mutations, has seen significant advancements in diagnosis, management, and treatment through next-generation sequencing (NGS) platforms [1]. These technological innovations have revolutionized genomics, offering insights into genome structure, function, and dynamics. Human and cancer genomes sequenced with NGS, particularly through initiatives like TCGA (The Cancer Genome Atlas) and the ICGC (International Cancer Genome Consortium), provide invaluable resources for understanding the molecular underpinnings of cancer across various types [2]. NGS encompasses multiple sequencing techniques such as whole-genome sequencing (WGS), whole-exome sequencing (WES), transcriptome sequencing, and targeted region sequencing. WGS covers the entire genome but requires substantial DNA samples and sequencing depth, while WES focuses on coding regions, reducing cost and time compared to WGS. RNA sequencing (RNA-Seq) allows detection of alternative gene-spliced transcripts, post-transcriptional modifications, gene fusion, mutations, and changes in gene expression. Despite having a multitude of applications, only WGS, WES, and sc-RNA seq have been extensively used in research as well as in clinics. Compared to traditional sequencing methods, NGS offers advantages such as high throughput, reduced turnaround time, low-input DNA/RNA requirements, and the ability to screen various genomic aberrations simultaneously with high accuracy and sensitivity. Commercially available NGS platforms of some notable companies, such as Illumina, Pacific Biosciences, Oxford Nanopore, Roche, Ion Torrent, and Life Technologies, each offer unique capabilities for cancer genome analysis [3]. These platforms have been pivotal in identifying disease-causing variants, revealing novel drug targets, and elucidating complex biological processes in cancer development. However, NGS faces challenges in clinical use due to issues such as sample selection, small sample sizes, and limited affordability for patients. Integrating various variant data analysis software with NGS can provide a comprehensive understanding of diseases like NSCLC, colorectal, ovarian, and leukemia, potentially enhancing cancer care and patient outcomes [4]. Through the integration of NGS technologies and powerful bioinformatics tools, researchers aim to decipher vast amounts of data to enhance our understanding of cancer biology and develop personalized treatment strategies. This review provides a comprehensive overview of NGS, and how its integration with current bioinformatics tools can enhance personalized oncology treatment.

## 2. Next-Generation Sequencing Techniques (NGS)

**Figure 1:**
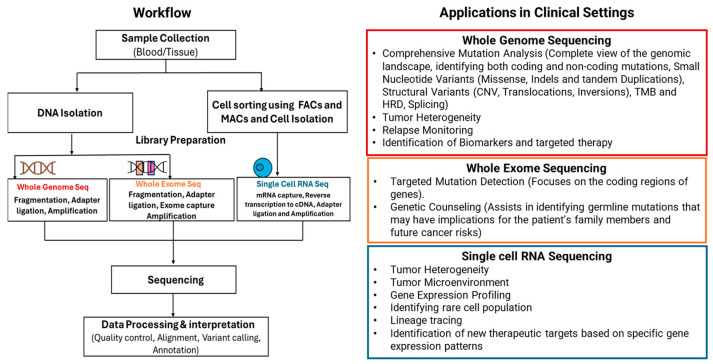
Workflow and clinical applications of WGS, WES, and sc-RNA-Seq.

The recent, remarkable progress in NGS technologies, coupled with advancements in bioinformatics and computational methodologies for handling massive datasets, allows clinicians to conduct in-depth analyses of numerous cancer genome profiles. This is achieved through sequencing techniques, such as WGS, WES, and sc-RNA-Seq [5]. Figure 1 illustrates the workflow and clinical applications of the various techniques discussed above and provides a comprehensive overview of their utility in practice.

### 2.1. Whole-Genome Sequencing (WGS)

WGS in cancer research offers a deeper understanding of the cancer genome landscape. This technique helps elucidate the functions of overlooked genomic regions by detecting underlying carcinogenesis and enabling molecular sub-classification of cancer [5]. In WGS, the short reads protocols mainly provide the coverage of 10× of 95% of the human genome and the median coverage of 30× [6]. WGS involves technically straightforward processes, where DNA is randomly fragmented and sequenced at a significant depth for both cancer and normal genomes. Two genomes per patient are sequenced—the germline from blood and the somatic from tumor samples. The analysis identifies single-nucleotide variants (SNVs), small indels, and structural variants (SVs). WGS surpasses existing diagnostic techniques in accuracy and accessibility, assessing various genomic biomarkers and mutational signatures. It outperforms hybridization probe-based methods by remaining up to date with emerging biomarkers and therapies, enabling reanalysis for new genetic associations, and providing germline data for cancer predisposition and pharmacogenomics [7]. WGS also highlights limitations in detecting complex structural rearrangements, prompting exploration of alternative methods like RNA sequencing. Additionally, it demonstrates potential in clinical trials for analytical accuracy and timely results, emphasizing the importance of defining clinically relevant thresholds for copy number alterations.

One of the major challenges in cancer WGS lies the reliability of WGS on fresh-frozen (FF) tissue samples due to their superior quality. FF samples are preferred because they preserve DNA integrity, minimizing fragmentation and chemical modifications. This high-quality DNA allows comprehensive genomic analyses, including structural variants and copy number alterations, making FF samples ideal for detailed investigations in clinical oncology. In contrast, the use of formalin-fixed, paraffin-embedded (FFPE) samples, which are more commonly available in clinical settings, presents several challenges, such as DNA fragmentation and chemical alterations caused by fixation processes. These factors can lead to artifacts, particularly T>C/G>A variants, and make them unsuitable for complex analyses. Additionally, FFPE samples tend to produce lower-quality libraries, compromising their reliability for comprehensive genomic studies [8]. Despite these limitations, there is a growing need for the potential value of FFPE samples in clinical genomics. To optimize WGS outcomes from FFPE tissues, several strategies have been proposed. Utilizing FFPE blocks that have been stored for shorter durations can help improve DNA quality. The application of uracil-DNA glycosylase treatment can mitigate deamination-related artifacts, while specialized extraction kits, such as the GeneRead DNA FFPE Kit, enhance both extraction and library preparation processes. Moreover, innovative techniques, like Safe-Seq, dual-barcoding, and circle sequencing, can reduce PCR errors and improve base calling accuracy. Implementing stringent quality control measures and employing advanced bioinformatics tools tailored to detect FFPE-related artifacts are crucial for generating reliable genomic data from these samples [9,10].

Recent studies underscore the clinical applicability of FFPE samples, introducing the FFPEimpact score to assist clinicians in interpreting WGS data. This score quantifies the extent of sequencing artifacts—specifically substitution and indel artifacts—allowing informed interpretation and better clinical decision making [11].

Another challenge is streamlining logistics by collecting and shipping blood and tissue samples together, alongside techniques like manual microdissection, which can maximize the utilization of available tumor material. These improvements ultimately enhance the accuracy and reliability of genomic analyses in clinical practice.

Additionally, handling large datasets in informatics and computational analysis requires substantial computational resources. The data produced by WES are about 30 GB, with corresponding variant files (.vcf) of about 1 GB, and are 24-fold higher than those of targeted and exome sequencing [6]. Commercially based cloud computing systems encounter issues like data transfer, ethics, and legal concerns, given the sensitive nature of genomic information [12]. Substantial investments in infrastructure, such as advanced instrumentation, skilled personnel, and high-performance computing hardware, are imperative. Sustaining this level of performance demands a considerable financial commitment, encompassing the acquisition and maintenance of sequencers, robust data storage solutions, and consumables. Achieving cost-effectiveness in this endeavor may necessitate the establishment of a large, well-funded, and centralized organization capable of managing the significant expenses associated with infrastructure. This includes not only the initial setup of sequencing facilities but also the ongoing operational costs, ensuring a sustainable and efficient utilization of resources for the successful implementation of WGS in clinical settings. However, full integration into clinical practice requires addressing clinical limitations, ensuring automated pipelines, and establishing robust ethical and regulatory frameworks. Despite the complexity and potential errors in WGS, its role in cancer research is crucial as it offers valuable insights into the genomic landscape and paves the way for personalized approaches in cancer diagnosis and treatment [13]. The genomic landscape of different cancer types using whole-genome sequencing is given in Table 1.

### 2.2. Whole-Exome Sequencing (WES)

WES is a robust NGS approach widely employed in cancer genomics, particularly for mutation discovery in cancers like melanoma. This technique involves sequencing all coding regions of the human genome, known as the exome, providing an unbiased and genome-wide screening strategy. Sequencing the complete coding regions (exome) holds promise for the uncovering of the causes of numerous rare, mainly monogenic genetic disorders, as well as the predisposing variants in common diseases and cancers [27].

Although WES misses noncoding regions, it offers improved sequencing coverage and depth for coding regions, enhancing sensitivity for detecting low-frequency mutations. The initial phase of WES involves the detection of single-nucleotide variants (SNVs) and copy number variations (CNVs). Variant calling algorithms for SNVs are employed, ranging from standalone tools to machine learning-based pipelines. CNV detection involves comparing the number of reads aligned to specific genomic segments. The integration of SNV and CNV data facilitates the identification of mutations with pharmacologically druggable alterations [28]. Notably, the variant calling and gene annotation of WES data reveal approximately 10,000 nonsynonymous variants per individual exome, with variations influenced by ethnicity and calling methods. This targeted approach pinpoints genetic variants that have an impact on proteins. Given that a substantial number of known disease-causing mutations are located within these protein-coding regions, exome sequencing emerges as a cost-effective method. Consequently, it presents a clinically viable approach for patient diagnostics by efficiently identifying relevant genetic variations.

In comparison to whole-genome sequencing, WES is favored for basic research and molecular diagnostics due to its comprehensiveness, efficiency, and cost-effectiveness. It has been instrumental in identifying recurrent mutations in melanoma [29]. WES aids in pinpointing homologous recombination deficiency, guiding the use of PARP inhibitors, and assessing microsatellite instability and the tumor mutation burden for selecting patients eligible for immunotherapy. WES offers the advantage of discerning low-frequency mutations that, when considered collectively, contribute to a nuanced and complex phenotypic presentation. WES provides insights into cancer mechanisms by comparing cancer cell DNA with normal cells, revealing germline and somatic mutations. Studies across cancer types use WES to detect driver mutations, enhancing knowledge of cancer pathways and aiding in therapeutic strategies, including pharmacogenetic variant identification. Intra-family WES can pinpoint predisposing variants and uncover cancer predisposition genes, further advancing cancer research and personalized medicine [27].

Despite its advantages, WES has limitations such as restricted CNV detection, lower coverage compared to targeted sequencing, and challenges related to references and minimal application requirements. The recognition of these limitations is crucial for advancing WES into clinical applications [28]. The genomic landscape of different cancer types using WES is shown in Table 2.

### 2.3. Single-Cell RNA Sequencing (sc-RNA-Seq)

Single-cell RNA sequencing (sc-RNA-seq) has emerged as a key tool in cancer research, significantly aiding in biomarker discovery, understanding cancer diversity, exploring drug resistance, studying the immune microenvironment, and improving immunotherapy. This technique allows researchers to detect differentially expressed genes (DEGs) and inter- and intratumor heterogeneity and to identify novel cell populations, gene expression, and the tumor microenvironment, thereby advancing immunotherapy and the identification of potential therapeutic targets [36]. By integrating genomics, transcriptomics, epigenomics, proteomics, and metabolomics, sc-RNA-seq offers a clearer view of transcriptome complexity with less background noise and a wider range for measuring RNA expression [37,38]. Table 3 represents the transcriptomic landscape of different cancers using sc-RNA sequencing. One of the main advantages of sc-RNA-seq is its ability to characterize individual cells, overcoming the limitations of bulk analyses. By examining tumor expression diversity, scientists can identify different cell types (like malignant cells, T cells, and fibroblasts) and more specific variations within these groups, known as ‘cell states’, which include differences in cell cycle and metabolic activity. Identifying these cell types and states involves analyzing genes that are expressed at varying levels, along with genetic features, such as copy number changes, point mutations, and fusion proteins [39].

The workflow of sc-RNA generally follows these steps: (1) isolating single cells, (2) extracting mRNA, (3) reverse transcription and cDNA amplification, (4) preparing the library, and (5) sequencing and analyzing the data. To isolate single cells, methods include cell selection, random dilution, laser microdissection (LCM), fluorescence-activated cell sorting (FACS), and microfluidic techniques. There are five main methods for sc-RNA sequencing: (1) Droplet-based 10× Genomics Chromium, (2) SMART-Seq, (3) the Tang method, (4) STRT-seq, and (5) CEL-seq [40]. Despite its promise, scRNA-seq faces several limitations, including the risk of damaging cell integrity and viability during isolation processes. The overall costs remain high due to the need to analyze large cell populations. Integrating scRNA-seq with other genomic data presents additional challenges. Moreover, effectively separating technical noise from biological signals is crucial for accurate analyses, highlighting the need for advanced computational methods [41]. Establishing quality control standards and addressing technical artifacts remain important, especially as data analyses become more complex in larger studies. The ongoing focus on sc-RNA-seq in tumor research underscores the need for collaborative data-sharing platforms. Centralized repositories are essential for handling large datasets, particularly in cancer studies, enabling easier access based on cell conditions rather than just sequence data. As bioinformatics and computational methods improve and the global sharing of sc-RNA-seq data increases, significant advancements in personalized medicine will become more attainable. Future efforts should emphasize refining bioinformatics pipelines, enhancing single-cell resolution technologies, and developing robust methods for comprehensive multi-omics analyses [37,42].

**Table 3:** Transcriptomic landscape of different cancer types using sc-RNA sequencing.

Cancer	Single cells	Transcriptomic Landscape	References
Breast Cancer	27,028 (primary tissue), 69,768 (axillary lymph nodes)	- **Breast Cancer Stem Cells (BCSCs)**: Identified as CD44+/ALDH2+/ALDH6A1+. - **Heterogeneity**: Inter- and intratumor variation linked to 103 gene downregulations. - **Metastasis Genes**: *PTMA*, *STC2*, *CST3*, *RAMP3*. - **CNV Clusters**: Cluster_4 showed high mutation rates associated with lymph node metastasis. - **Immune Interactions**: NECTIN2-TIGIT interactions promote immune escape. - **Key DEGs in TNBC**: *B2M*, *CD52*, *PTMA*, *GZMK*.	[43]
Lung Cancer	220,716	- **Heterogeneity**: Distinction between AT2 and basal cell types. Fibroblast and NE key cell types that distinguish two tumor subtypes from their adjacent tissues. - **Key Driver Genes**: *EGFR*, *KRAS*, *BRAF*, *ERBB2*, *MET*. Potential Therapeutic Targets: Specific subclones of AT2 and basal cells. - **Prognosis**: Better PFS and ORR with targeted therapies.	[44]
Pancreatic Ductal Adenocarcinoma (PDAC)	6236	- **Heterogeneity**: Notable intertumor variation.Tumorigenicity: Cancer stem cells as primary drivers. - **Pathways**: Enrichment in IL6/JAK/STAT3, PI3K/AKT/MTOR, TGF-β signaling. Gene expression: High expression during PDAC (EPCAM, KRT19, MUC1, CEACAM6). - **Key Drivers**: VEGF/VEGFR, HIF2, and P53 signaling pathway, MMP7, TSPAN8, MSLN, LAMC2, KLK6, and LY6D. - **Genes Involved in Tumor Progression**: *MUC1* and *CEACAM6*.	[45]
Colorectal Cancer (CRC)	9120	- **DEGs**: Lower expression of enterocyte (CA1, CA2) and endocrine markers (PYY, GCG); metallothionein family genes (*MT1H* and *MT1G),* higher expression of *LY6E, FXYD5, TGFBI*. - **Metastasis**: Upregulation of EKC/KEOPS. - **Key Mutations**: *KRAS* mutations in actively dividing tumors. - **Potential Therapeutic targets**: PPAR inhibitors, WNT inhibitors.	[46]
HBV-associated Hepatocellular Carcinoma (HCC)	>1000	- **Heterogeneity**: Intertumor heterogeneity more prominent than the intratumor due to the cells clustering together according to similarities of global transcriptomic profile, LCSC markers, inferred CNV status, and RTK expression. - **Prognosis**: Poor outcomes linked to high TAM markers. - **Drug Resistance**: Intratumor heterogeneity leads to resistance against RTK inhibitors. - **Potential Therapeutic Targets**: TIGIT–NECTIN2 axis.	[47]
Acute Myeloid Leukemia (AML)	91,772	- **Potential Targets**: Enhanced interaction between HLA-F, HLA-E, HLA-C, and B/CD8 + T/HSC-Prog/plasma in NK cells. - **Recurrence Risk**: Associated with CD4^+^ Tregs. - **Gene Expression**: Overexpression of inflammatory response genes, CD14^+^ monocytes, hyperactive BATF. - **Signaling Pathways**: Increased activity in T cell subsets of CD4^+^ and CD8^+^ T cell signaling pathways related to TNFA, NFKB, hypoxia, KRAS, MTORC1, and other hallmark gene sets in AML patients using GSVA and GSEA. - **Progression**: Associated with an increase in the number of CD14^+^ monocytes and monocyte-DCs as the CNVs changed. - **Heterogeneity**: CNV and intercellular interaction networks in HSC-Prog cells. HSC-Prog exhibits great heterogeneity in chromosomal structure.	[48]

## 3. NGS for Personalized Oncology

NGS holds the added advantage of providing a more comprehensive picture of the cancer landscape and uncovering cancer development pathways.

### 3.1. Tumor Heterogeneity

Cancer development involves the gradual accumulation of somatic genomic alterations during clonal evolution, creating opportunities for selective advantages and intratumor heterogeneity [43]. Tumor heterogeneity presents substantial obstacles in the clinical understanding and management of cancer. Differences can emerge even among tumors sharing the same histologic subtype, leading to diverse therapeutic outcomes in patients. The intricate nature of tumor heterogeneity significantly impacts responses to treatment, disease recurrence, and, ultimately, patient survival [49]. For instance, in gastric adenocarcinoma, Wong et al. (2014) used WGS to uncover intricate intratumor heterogeneity in *TP53* inactivation mechanisms. Two distinct processes, involving *TP53* mutations with copy loss and homozygous loss, were observed within the same tumor [50]. Similarly, Morrison et al. (2014) noted prevalent *TP53* mutations in bladder cancer, which contributed to increased nucleotide-level intertumor heterogeneity due to early clonal expansion and insufficient DNA repair processes [51]. Zhang et al., 2013 addressed the significance of intratumor genetic heterogeneity using WGS, particularly in the context of treatment resistance and the potential development of targeted therapies for resistant and metastatic tumor cells in head and neck squamous cell carcinoma. Notably, only a slight majority of the genes with somatic point mutations were shared across all the tumor samples, underscoring the significance of understanding intratumor heterogeneity for accurate biomarker identification. The degree of heterogeneity enabled the estimation of clonal expansion and a timeline for tumor development, revealing a branching evolutionary process with potential implications for early tumor detection [52]. Leong et al. (2019) emphasized CNV as a major influencer of genomic heterogeneity in lung cancer [53], but Ishaque et al. (2018) proposed additional mechanisms involving chromosome 4 amplifications and the roles of *PDGFRA*, *KIT*, *KDR*, and *REST* in promoting proliferation and suppressing metastasis. Notably, *PDGFRA* and *KDR* play crucial roles in enhancing metastatic potential, highlighting the complex interplay driving tumor heterogeneity [54]. In epithelial ovarian cancer, Lee et al. (2015) revealed the late-stage divergence of metastatic clones and primary tumor clusters in both ovaries, demonstrating diverse intratumor heterogeneity [55]. Ho et al. (2021) employed scRNA-Seq in HBV-associated hepatocellular carcinoma (HCC) patients, revealing significant intra- and intertumor heterogeneity. The co-existence of intra- and intertumor heterogeneity, particularly the presence of rare subclones, may contribute to the failure of targeted therapies in HCC [47]. Similarly, Wu et al. (2017) utilized single-cell WES in colorectal adenoma and cancer samples, revealing their monoclonal origins, shared mutations in signaling pathways, and the emergence of intratumor heterogeneity, with nonrandom mutations accumulating in the GPCR, PI3K-Akt, and FGFR pathways [56]. Xu et al. (2021) explored breast cancer lymph node metastasis through scRNA-Seq, revealing inter- and intratumor heterogeneity [43]. Bao et al. (2020) focused on triple-negative breast cancer (TNBC), identifying extensive heterogeneity at the single-cell level, with a subset of cells expressing epithelial–mesenchymal transition (EMT), stemness, and angiogenesis, which potentially contribute to TNBC aggressiveness [57].

All these high-resolution approaches not only aid in unraveling the molecular underpinnings of treatment resistance and disease progression but also facilitate the discovery of novel biomarkers and therapeutic targets. Consequently, NGS has become indispensable in the quest to tailor personalized treatment strategies that can effectively address the challenges posed by both inter- and intratumor heterogeneity, ultimately improving patient outcomes in the clinical setting.

### 3.2. Targeted Therapy

Targeted therapies transform cancer treatment by focusing on specific molecules and proteins involved in tumor growth. Advances in genetic testing and NGS allow the identification of unique genetic abnormalities, enabling personalized treatment strategies. For example, *KRAS* mutations, which are present in 33% of colorectal cancers, play a crucial role in early progression to carcinoma, impacting treatment decisions and rendering certain therapies ineffective [58]. Similarly, *BRAF*, *NRAS*, and *KIT*, in the case of melanoma, have led to the development of targeted therapies, which are particularly effective against tumors harboring BRAF mutations [59]. Drug resistance is frequently associated with the mTORC1 pathway, which is vital for cell metabolism and growth, suggesting that targeting this pathway could enhance treatment efficacy [60]. In the case of non-small cell lung cancer (NSCLC), *TP53* mutations are common and linked to aggressive disease and poor outcomes [61]. These mutations, along with changes in other tumor suppressor genes, like *NF1* and *RB1*, complicate treatment. Targeting these pathways offers new therapeutic options, such as gene therapies using p53 and MDA-7/IL-24 to induce cancer cell death. There is also potential for research focusing on kinase inhibitors and the inhibition of p73-dependent growth [62]. WES has shown a genetic landscape in pancreatic carcinoma, which suggests potential therapeutic targets, including *BRAF* and *PIK3CA* mutations, alterations in DNA repair, and chromatin remodeling pathways [63]. Additionally, the carcinoma of unknown primary site (CUP) samples showed clinically relevant genomic alterations for personalized treatment strategies, particularly in the RTK/Ras/mitogen-activated protein kinase pathway [64]. Using NGS, inflammatory breast cancer (IBC) exhibited clinically significant genetic alterations (CRGA) in genes like *TP53*, *MYC*, *ERBB2*, *FGFR1*, *BRCA2*, and *PTEN.* This information guides personalized treatment, transitioning from traditional chemotherapy to more targeted therapies, potentially enhancing efficacy and minimizing side effects [65]. In clinically advanced prostate tumors, NGS identified genetic alterations in key pathways, highlighting potential biomarkers for targeted therapies in androgen axis inhibitor-resistant tumors [66]. In a study by Bahceci et al. (2023) on craniofacial ossifying fibromas (OF), recurrent focal copy number gains and pathogenic mutations in the *CDC73* gene were identified, showing the rarity of MDM2 amplification in OF, which is crucial for distinguishing low-grade osteosarcoma from OF and emphasizes the role of activator protein 1 (AP-1) transcription factors in the pathogenesis of juvenile trabecular OF [67]. In gastric cancer, *RHOA* mutations suggest targeted therapeutic interventions [16], while in papillary thyroid cancer, *EML4–ALK* translocations and *TRAPP* oncogene mutations provide opportunities for personalized treatment [68]. These studies collectively emphasize the utility of NGS in uncovering clinically relevant genetic alterations to inform targeted therapeutic strategies in diverse cancer types.

### 3.3. Resistance Mechanisms

The challenge of overcoming resistance to cancer drugs persists due to the intrinsic heterogeneity within genetically unstable tumors. Addressing this complexity necessitates a holistic understanding of genetic, epigenetic, transcriptomic, and proteomic modifications [69]. NGS can help identify mutations that lead to drug resistance. This information can guide the selection of alternative treatments or combination therapies to overcome resistance. For instance, in breast cancer patients, WES has uncovered the potential mechanisms underlying resistance to Herceptin and tyrosine kinase inhibitor (TKI) therapies. Specifically, high C>T mutations, significant differences in transition-to-transversion (TiTv) ratios, and microsatellite instability-high (MSI-H) status may indicate resistance to Herceptin. Conversely, a similar mutation profile with the absence of MSI-H may suggest resistance to TKIs. Moreover, mutations in APOB are found in patients resistant to both Herceptin and TKI treatments [70]. Similarly, Turajlic et al. (2014) explored the intricate genetic landscape contributing to drug resistance in melanoma patients exhibiting inherent resistance to vemurafenib. The study also revealed the potential efficacy of a combined approach utilizing AKT and MEK inhibitors. Notably, the presence of *BRAFV600E*, coupled with a GNAQ mutation (A>C, p.Q209P) and a *PTEN* frame-shift deletion, was linked to heightened AKT activity, supporting a promising, synergistic treatment combination capable of blocking tumor growth [71]. In a parallel pursuit, Patch et al. (2015) elucidated mechanisms of chemoresistance in recurrent, high-grade serous carcinoma (HGSC). Fusion events in *ABCB1* were identified, correlating with elevated expression and drug resistance. The study also shed light on the reversion of BRCA1 and BRCA2 germline alleles as a potential pathway for resistance evolution. Molecular subtype switching and stromal reactions were implicated in the influencing of drug resistance in HGSC [72]. Ross et al. (2013) observed an unexpectedly high frequency of genomic alterations that influenced targeted therapy selection for HGSC. Notably, TP53 mutations were present in over 85% of high-grade serous carcinomas, while *NF1* mutations were found in 14% of cases, exclusively in tumors positive for *TP53* mutation. Due to NGS, the identification of alterations in the mTOR/ PI3K pathway and members of the *EGFR* family suggests potential clinical utility for inhibitors of the mTOR/PI3K pathway [73].

Miyamoto et al. (2015) contributed to the understanding of acquired drug resistance in castration-resistant prostate cancer (CRPC) patients treated with enzalutamide, an androgen receptor (AR) inhibitor. Single-cell RNA sequencing of circulating tumor cells (CTCs) highlighted heterogeneity among the CTCs within and across patients, indicating differences from the primary tumor specimens. The study suggested that noncanonical Wnt signaling is implicated in antiandrogen resistance, potentially acting through an alternative pathway to AR abnormalities [74].

Wang et al. (2020) employed scRNA sequencing to investigate mantle cell lymphoma (MCL). Their findings revealed high cellular heterogeneity and a common origin for identified cell clusters. Malignant B cells, especially type I B cells, exhibited increased proliferation, implying a role in immune escape and drug resistance [37]. These studies collectively emphasize the critical role of NGS in pharmacogenomics for the treatment of various cancers. The integration of these findings into therapeutic strategies holds promise for more effective cancer interventions.

### 3.4. Prognosis and Predictive Biomarkers

NGS technologies are an indispensable tool for refining prognostic assessments and tailoring therapeutic strategies in the complex landscape of cancer. Itamochi et al. (2017) highlighted the significance of WGS in predicting the prognosis for ovarian clear cell carcinoma (OCCC). Frequent mutations in *ARID1A* and *PIK3CA* and the activation of the PI3K/Akt and RTK/Ras signaling pathways could serve as favorable prognostic indicators for OCCC patients [75]. The integration of NGS with liquid biopsies, which analyze circulating tumor DNA (ctDNA), provides real-time insights into tumor dynamics, enabling early cancer diagnosis and the monitoring of treatment responses. A study by Marchetti et al. (2014) integrated NGS with liquid biopsies to monitor *EGFR* mutations in NSCLC patients. There were *EGFR* mutations in 84% of the analyzed patients, demonstrating the effectiveness of CTC analysis for real-time mutation detection and its potential in personalizing treatment [76]. Zhang et al. (2023) employed NGS techniques for both circulating tumor DNA (ctDNA) testing and tissue sequencing; the researchers detected mutations in key genes, such as *TP53*, *RB1*, and *PTEN*, and the dynamic somatic mutation profile of extensive-stage small-cell lung cancer (ES-SCLC). The correlation between ctDNA levels and tumor burden, identified through NGS, emerged as a crucial prognostic indicator, providing real-time information on disease progression and treatment response [77]. Yamada et al. (2018) identified novel dysregulated long noncoding RNAs (lncRNAs) in colorectal cancer. Among these, CRCAL-3 and CRCAL-4 were highlighted for their functional significance in cell cycle regulation. The study suggests that these lncRNAs, particularly CRCALs, could potentially serve as early biomarkers for colorectal cancer detection [78].

Ho et al. (2012) compared liver cancer gene expression using RNA-Seq, revealing the upregulation of inflammation, chemoresistance, and lipid metabolism genes in CD90+CSCs. Lipid metabolism and specific genes, like *APOE*, *APOC1*, *ESM-1*, *PLVAP*, and *GPC3*, in CD90+CSCs suggested roles in proliferation, angiogenesis, and potential therapeutic targets [79].

### 3.5. Identification of Driver Mutations

Cancer cells undergo numerous genetic changes, but only a subset, known as driver mutations, plays a significant role in cancer progression. Identifying these driver mutations can be challenging due to the high diversity of mutations and tissue types in tumors [80]. However, recent studies have highlighted the potential of NGS platforms to detect known driver mutations that are clinically relevant for guiding treatment decisions. In a study by Cifola et al. (2013), WES and SNP array profiling on six melanoma cell lines from metastatic patients confirmed well-known melanoma driver mutations, such as *BRAFV600E* and *NRASQ61R*, while also discovering novel mutations in genes tied to critical signaling pathways involved in melanoma, including the MAPK and glutamate pathways [29]. The identification of these driver mutations using NGS is particularly crucial in addressing the genetic and environmental variations across different ethnic groups and geographic regions, which contribute to disparities in cancer risk and treatment outcomes. For instance, variations in driver mutations can differ among populations due to genetic diversity, while environmental factors like diet, healthcare policies, and economic disparities further influence these differences. One example is the CGP study, which utilized NGS in tumor samples from 306 Chinese lung cancer patients. This study revealed significant differences in driver mutations between Asian and Caucasian populations. In the Chinese cohort, the most frequently mutated genes were *EGFR*, *TP53*, *ALK*, and *KRAS*. Compared to the dataset of The Cancer Genome Atlas (TCGA), *EGFR* mutations were more common in the Chinese group, while *KRAS* mutations were found in only 9.8% of the Chinese adenocarcinoma patients, significantly lower than in Caucasians. The NGS approach identified clinically actionable alterations in 7.2% of the patients that routine tests missed, highlighting the potential for more targeted treatments. These alterations often involved genes like *PIK3CA*, *ROS1*, and *MET*, some of which were linked to resistance to *EGFR* TKIs [81]. Similarly, another study identified significant variations in polygenic risk scores (PRS) across diverse ancestry groups, including Arabs, Persians, and South Asians. This variation advocates for personalized cancer strategies while also raising ethical concerns around genetic testing. Individuals from the Arabian Peninsula had the lowest mean PRS for colorectal cancer, while those of African ancestry had the highest for prostate cancer. Interestingly, no individuals of Arabian Peninsula origin carried known breast or ovarian cancer variants, suggesting a potentially lower genetic risk in this subgroup. In contrast, variants associated with breast cancer were prevalent among Qataris of Persian origin [82]. These studies highlighted the need for an inclusive approach to cancer research and treatment that considers ethnic and population-specific genomic differences. By integrating genetic insights with socioeconomic factors, we can better address health disparities in cancer outcomes.

## 4. Data Analysis and Bioinformatics Tools

The exponential growth of data generated by NGS necessitates advanced computational and bioinformatics expertise for effective management, analysis, and interpretation. This increase in data has led to significant advancements in NGS bioinformatics, driven largely by improvements in computational hardware and the development of sophisticated algorithms and software applications. These advancements support all stages of data processing, from initial raw data handling to in-depth analysis and clinical variant interpretation [83]. NGS bioinformatics is typically divided into three main analysis phases: primary, secondary, and tertiary. While the overarching goals of these analyses remain consistent across different NGS platforms, each platform—particularly the two leading commercial second-generation systems, Illumina and Ion Torrent—has its own unique features and requirements. The computational analysis of NGS data comprises several critical steps, including the processing of raw sequencing data, mapping reads to the human reference genome, post-processing alignments, and variant calling. Each step is essential for obtaining meaningful insights from the data. However, there is considerable analytical diversity in the tools employed, whether they are published methods or proprietary solutions, as well as in their specific parameter settings. This flexibility enables researchers to customize their analyses to suit the unique characteristics of their datasets, including the incorporation of cancer-specific variant calling and annotation [84]. These bioinformatics resources play a crucial role in enhancing our understanding of the molecular mechanisms underlying tumorigenesis and progression, while also aiding in the identification of potential therapeutic targets. By using these tools, researchers can accelerate the discovery of insights that are vital for advancing cancer treatment and improving patient outcomes [85]. Table 4 summarizes these computational tools used by various studies for genome profiling in cancer treatment.

Recently, artificial intelligence (AI) has been playing an increasingly significant role in personalized medicine, due to the increasing availability of large cancer datasets. With deep learning (DL) techniques, AI is emerging as an impressive tool for biomedical data analysis. The integration of multi-omics data with clinical information enhances biomarker identification and prognostic accuracy, facilitating a more nuanced understanding of cancer [91]. AI algorithms can synthesize data from diverse platforms, including genomics, epigenomics, transcriptomics, proteomics, metabolomics, pathomics, and radiomics, enabling precise identification of cancer subtypes. This multifaceted approach provides robust tools for predicting cancer prognosis and treatment responses [92]. For instance, Champion et al. (2018) developed AMARETTO, an algorithm that integrates multiple molecular data sources to identify a network of pan-cancer driver genes through the combination of copy number variations, DNA methylation, and gene expression data [93]. Yuan et al. (2022) demonstrated the potential of a deep learning model, ResNet3D + SVM classifier, for predicting peritoneal carcinomatosis in colorectal cancer [94]. In the context of triple-negative breast cancer, Azzouz et al. (2021) employed a machine learning algorithm to address treatment heterogeneity among subtypes [95]. Additionally, Jiao et al. (2020) utilized a deep learning classifier to distinguish 24 major tumor types based on patterns of somatic mutations detected through WGS, achieving impressive accuracy rates of 91% on held-out tumor samples and 88% and 83% on independent primary and metastatic samples, respectively [96]. For whole-exome sequencing, Sun et al. (2019) introduced the genome deep learning (GDL) method, employing a deep neural network model to identify cancer risk by analyzing genomic variations. This GDL model effectively differentiates between 12 types of healthy and cancerous tissues based on point mutations in WES data [97]. Moreover, Zhang et al., 2023 analyzed the bulk of scRNA sequencing data, by using the tumor-infiltrating immune cell (TIIC)-associated signature based on a total of 26 machine learning (ML) algorithms. The TIIC signature score showed superior performance to 168 previously established signatures in lung adenocarcinoma and showed a prognostic value that can forecast genomic change, chemotherapeutic drug susceptibility, and—most significantly—immunotherapeutic response [98]. Despite these advancements, challenges remain in the field of AI in oncology. Issues such as data heterogeneity, the need for standardization across datasets, and the interpretability of complex models pose significant hurdles. Furthermore, ethical considerations regarding patient privacy, data security, and algorithmic bias must be addressed to ensure equitable outcomes [91,92].

## 5. Challenges and Opportunities

The integration of NGS into cancer treatment presents significant challenges and controversial findings that complicate its clinical application. For instance, the SHIVA trial highlights the limitations of off-label targeted therapies, which failed to demonstrate a clear benefit over standard treatments when solely relying on molecular alterations [99]. Additionally, tumor heterogeneity presents challenges; both intertumor and intratumor variability complicate biomarker identification, which is crucial for developing and administering molecular targeted therapies based on single tumor biopsy specimens. The use of circulating tumor cells (CTCs) or circulating tumor DNA (ctDNA) could potentially address this issue by providing a broader perspective on overall tumor heterogeneity [100,101]. Sample quality also poses substantial obstacles to the successful application of NGS, as smaller specimens often lead to failures due to insufficient tumor content or compromised DNA quality. The sensitivity of NGS for SNVs is around 5% to 10% [102,103,104], and FFPE samples often show higher artifact rates compared to fresh tissues, with systematic error rates of 4% to 6% [105]. A study by Al-Kateb et al. (2015) investigates the factors affecting NGS success in cancer tissue specimens, revealing that 22.5% of 1528 analyzed samples failed, primarily due to pre-analytical issues such as insufficient tissue (INST) and low DNA integrity (INS-DNA), which accounted for about 94% of the failures. The key factors contributing to INST failures include shorter time from tissue collection to NGS analysis, higher tumor heterogeneity, and lower viability. The findings underscore the importance of larger tissue samples for effective analysis [106]. Techniques like overlapping paired-end reads [107] and random nucleotide tags (unique identifiers or UIDs) [108] have been developed to improve variant detection accuracy and sensitivity. While NGS performs well for SNVs and small indels, its effectiveness for structural variants (SVs) and copy number variations (CNVs) is less robust, particularly in repetitive genomic regions [109,110]. CNV analyses can yield false positives ranging from 10% to 89% [111], underscoring the need for refined detection methods. Access to clinical trials poses another hurdle; strict eligibility criteria and geographical limitations often prevent many patients from participating in potentially beneficial studies; this creates significant challenges in the gathering of sufficiently large populations to conduct randomized controlled trials for each cancer subtype identified by NGS. The first precision medicine randomized controlled trial indicated no significant improvement in patient outcomes when therapies were matched to molecular profiles. Therefore, the integration of biomarkers, molecular tests, and targeted drugs necessitates concurrent development and thorough investigation to realize the full potential of personalized medicine [112]. Moreover, the oncologists’ comfort and familiarity with interpreting genomic data, alongside patient preferences and financial considerations, can limit the potential benefits of NGS. Actionable mutations identified through NGS do not consistently lead to corresponding therapies, with only a small percentage of patients receiving treatments tailored to their molecular profiles. Various databases exist for genomic data, but they often lack comprehensiveness and accuracy, especially regarding rare variants [113,114]. Integrating genomic data into clinical practice presents challenges for smaller healthcare institutions, requiring updates to operational processes, training, and technology. This complexity is aided by large publicly accessible databases, such as COSMIC (Catalogue of Somatic Mutations in Cancer), the UCSC Cancer Genomics Browser, and cBioPortal [115].

Despite these challenges, the positive aspects of NGS deserve recognition. The MOSCATO 01 trial found that high-throughput genomic analyses identified actionable alterations in a significant proportion of patients, with some experiencing improved progression-free survival [116]. These findings suggest that, while NGS faces numerous hurdles, it holds promise for the enhancement of treatment strategies in select patient populations. Addressing the multifaceted barriers surrounding NGS implementation—ranging from regulatory policies to insurance coverage—is essential for unlocking its full potential in precision medicine and improving outcomes in oncology.

## 6. Conclusions

NGS has emerged as a transformative tool in cancer research, offering unparalleled insights into the genomic landscape of the disease. By detecting previously unexplored mutations and providing a comprehensive understanding of genomic alterations, NGS facilitates the molecular sub-classification of cancer and enhances our knowledge of underlying carcinogenesis. Despite its technical complexities and computational demands, NGS outperforms traditional diagnostic techniques in accuracy, enabling the identification of various genomic biomarkers and mutational signatures. However, challenges such as availability and financial investments remain significant hurdles in the widespread adoption of NGS in clinical settings. Achieving cost-effectiveness and seamless integration into clinical practice requires a concerted effort involving the establishment of robust ethical and regulatory frameworks, automated analysis pipelines, and sustained financial support. Despite these challenges, the role of NGS in cancer research cannot be overstated. Its ability to provide valuable insights into the genomic landscape of cancer holds promise for personalized approaches in cancer diagnosis and treatment. Continued advancements in technology, bioinformatics, and data analysis methodologies will further enhance the utility of NGS, ultimately improving patient outcomes in the fight against cancer.

## Figures and Tables

**Table 1:** Genomic landscape of different cancer types using whole-genome sequencing.

Cancer Type	Sample Size	Mutation Frequency	Gene Alteration Pathway	Mutational Signatures	Predictive Biomarker	References
Parathyroid carcinoma	23	*CDC73* (39.1%)	PI3K/AKT/mTOR (78.3%)	*CDC73* mutant group: signatures 1, 2, 3, 9, and 13; wild-type *CDC73*: 1, 3, 5, 9, 16, 28, and 30		[14]
Triple-negative breast cancers (TNBCs)	254	*BRCA1*, *BRCA2*, *PALB2*, *RAD51C* (67%)	PIK3CA/AKT1 pathway abnormalities (4.7%)	Mutational signatures of a PALB2 biallelic altered TNBC; RAD51C hypermethylated TNBC		[15]
Gastric cancer	100	*RHOA* (14.3%) diffuse-type tumors	Adherens junction pathway, focal adhesions pathways			[16]
Lung adenocarcinoma	230	RIT1-activating mutations, loss-of-function MGA mutations, *EGFR* mutations (in female), *RBM10* (in males), *F1*, *MET*, *ERBB2*, and *RIT1* occurred in 13%	MAPK and PI(3)K pathway			[17]
Metastatic colorectal cancer	429	*LINC00672* mutations and 10 kb–1 Mb deletions. *FBXW7* (11.9%) *TP53* (73.9%); *KRAS* (47.3%), *APC* (78.3%); *PIK3CA* (15.9%); *ZFP36L2* (9.8%)	-	SBS1, 8 and 41, as well as DBS2, 4, and 6, SBS9/39/41, polymerase Pol η (associated with therapy resistance).	*FBXW7* mutations as a predictive biomarker for poor response to *EGFR*-targeted treatment	[18]
Pancreatic cancer	100	*TP53* (74%), *SMAD4* (31%), *CDKN2A*(35%), *ARID1A*, and *ROBO2*. *KDM6A* (18%) and *PREX2* (10%), *RNF43* (10%),	*BRCA* pathway	Top quintile of the BRCA signature	Mutations in *BRCA* pathway component genes and surrogate measures of defects in DNA maintenance (genomic instability and the *BRCA* mutational signature)	[19]
Ovarian clear cell carcinoma	15	*PIK3CA* (40%), *ARID1A* (40%), and *KRAS* (20%). Copy number gains in *NTRK1* (33%), *MYC* (40%), and *GNAS* (47%) and copy number losses in *TET2* (73%), *TSC1* (67%), *BRCA2* (60%), and *SMAD4* (47%)	PI3K/AKT, TP53, and ERBB2 pathways in 87%. Chromatin remodeling in 47% of OCCCs		ATR inhibitors	[20]
Bladder cancer	65	Mutated protein-coding genes: *ZFP36L1* (12.3%), *ELF3* (9.2%); noncoding mutations: *ERT*, *ADGRG6*, *PLEKHS1*, *WDR74*, and *LEPROTL1* (63%).	*HRAS/KRAS*, *PI3K*, *FGFR1/FGFR3*, *FAK*, *MTOR*, and *PKCB/PKCG* were altered in 23%, 22%, 17%, 8%, 7%, and 7% of the tumors, respectively	Signature D (8, 4, and 31), which was enriched C>A and T>A substitutions	Mutation in ADGRG6 enhancer	[21]
Cervical cancer	102	*PIK3CA* (16.7%), *FBXW7* (12.8%), *MLL3* (7.8%), *CASP8* (3.9%), and *FADD* (3.9%); *FAT1* (8.8%), *MLL2* (5.9%), and *EP300* (5.9%).	RTK/RAS/PI(3)K, cell cycle, and apoptosis pathways were altered in 88%, 74%, and 73% of cases, respectively	APOBEC family member APOBEC3H was expressed at higher levels in CC	The combination of HPV integration and DNA testing had a trend towards higher AUC value than HPV DNA, suggesting a better biomarker for cervical cancer screening.	[22]
Papillary renal cell carcinoma	169	In patients with primary tumor tissue *MET* (33%), *TERT* (30%), *CDKN2A/B* (13%), and *EGFR* (8%). In patients with metastatic tissue *CDKN2A/B* (18%), *TERT* (18%), *NF2* (13%), and *FH* (13%); *MET* (7%).	SWI/SNF complexes (26%), chromatin modification (24%), and cell cycle regulation (22%). RAS/RAF pathway (7%), PI3K/mTOR pathway (8%), and DNA damage pathway (8%)	-	MET, CKDN2A/B, and SWI/SNF pathway	[23]
Liver Cancer	300	Protein-altering mutations: *TP53*, *CTNNB1*, *ARID2*, *ARID1A*, *RB1*, *AXIN1*, *RPS6KA3*, *SETDB1*, *NFE2L2*, *BAP1*, and *HNF4A*; loss-of-function mutations: *ARID2*, *ARID1A*, *AXIN1*, *TP53*, *BRD7*, *RPS6KA3*, *RB1*, and *HNF4A*; mutations in the noncoding region *NEAT1* (22%) and *MALAT1* (6%)		Signature W1 (age-dependent); signature 4 (presence of TP53 mutations, smoking status, co-occurrence of the liver cancer with bladder or ureter cancer); signature W5 (alcohol intake); signature W2 (mutations in ARID family members); signatures W3 and W5 (presence of TERT promoter mutations); signatures W4, W6, and W7 (strong correlations with dinucleotide substitution); W4 and W6 (CC>AA substitutions).		[24]
Hepatocellular carcinoma (HCC)	254	*RB1* (11%), *ARID1A* (10%), *AXIN1* (9%), *ARID2* (8%), *TERT* (47.24%)		Signature 1 (19.29%, SBS22 associated with the plant-derived carcinogen aristolochic acid (AA) with a predominance of A:T-to-T:A transversions at T/CAG tri-nucleotide motifs. Signature 2 SBS5 with unknown etiology. Signature 3 SBS9 and associated with polymerase eta.	CNAs, SVs, expression levels, alternative transcripts, and fusion transcripts	[25]
AML	305	RAS/RTK Pathway Mutations: *(63%) KIT* (27%), *NRAS* (14.8%), *FLT3* (16.9%; 10% of all patients harbored an *FLT3*-ITD), *KRAS* (5.7%), and *CBL* (5%) epigenetic regulation (45%): *ASXL2* (15.7%), *ASXL1* (12.4%), *TET2* (7.9%), *EZH2* (5.7%), and *KDM6A* (4.2%)	RAS/RTK signaling pathways	-	*JAK2* mutations *FLT3-ITD* high, *KIT* mutations Therapeutic targets Midostaurin, Dasatinib, and other RTK inhibitors	[26]

**Table 2:** Genomic landscape of different cancer types using whole-exome sequencing.

Cancer	Sample Size	Mutation Frequency	Gene Alteration Pathway	Mutational Signatures	Predictive Biomarker/Therapeutic	References
Melanoma	8	*BRAF, NRAS*, and NF1. Heterogeneous somatic mutations 3–38%.	MAPK pathway	UVB-induced C>T transitions	ITH may be a prognostic biomarker	[30]
Ovarian clear cell carcinoma (OCCC)	15	*PIK3CA* (40%), *ARID1A* (40%), and *KRAS* (20%); *NTRK1* (33%), *MYC* (40%), and *GNAS* (47%); *TET2* (73%), *TSC1* (67%), *BRCA2* (60%), and *SMAD4* (47%).	PI3K/AKT, TP53, and ERBB2 pathways	-	-	[20]
Low grade serous ovarian carcinoma (LGOS)	63	Canonical MAPK mutant (cMAPKm: 52%, *KRAS*/*BRAF*/*NRAS*), MAPK-associated gene mutation (MAPK-assoc: 27%), and MAPK wild-type (MAPKwt: 21%).	NOTCH pathway	COSMIC signature SBS1, which is associated with aging and signature SBS10b, associated with elevated TMB.	Signature SBS10b, a potential biomarker	[31]
Breast Cancer	16	*KMT2C* (42%) followed by *HECTD1*, *LAMA3, FLG2, UGT2B4, STK33, BRCA2, ACP4*, *PIK3CA*, and *DNAH8* (33%).	PI3K/AKT/mTOR pathway, hyperactivation of the IL-6 pathway	C>T transitions; mix of C>G and C>T transitions	-	[32]
Pancreatic Cancer	21	*KRAS* (100%), *TP53* (74%), *CDKN2A* (16%), and *SMAD4* (10%).	*KRAS* signaling, TGF-β signaling, chromatin remodeling, Wnt signaling, DNA damage repair, cell cycle, and RNA processing	-	Presence of RNF43 mutations	[33]
Inflammatory Bowel Disease−Associated Colorectal Cancers	31	*TP53* (63%), *APC* (13%), and *KRAS* (20%).	WNT pathway	C:G>T:A at CpG	-	[34]
AML with abn(7)	60	*TP53* (33%), *NF1* (20%), *RUNX1* (20%), and *DNMT3A* (18.3%), *DNMT3A* (18.3%), *ASXL1* (11.7%), *TET2* (11.7%), *IDH2* (10%), *KMT2C* (10%), *EZH2* (8.3%), and *IDH1* (8.3%).		Sig-A, with high cosine similarities to SBS1/SBS5 in COSMIC (0.939) and SBS1/SBSblood in normal blood cells (0.945)		[35]

**Table 4:** Computational tools used for data analysis for genome profiling in cancer treatment.

Computational Tools/Web Servers/Databases	Description	References
FastQc	To assess the quality of sequencing runs	[28,84,86,87,88,89]
Alfred and Qualimap	To assess the mapping quality	
Bwa-mem; Bowtie2	Alignment of raw reads to reference genome	
STAR and HISAT	Aligners for RNA sequencing data	
minimap2	Aligner for mapping long-read sequencing	
SAMtools (v. 1.3.1), mpileup, and platypus	Manipulation in the SAM/BAM/CRAM format	
MuTect, VarScan2, SomaticSniper, Strelka, and FreeBayes, SigMA, CHORD, PathAI, AcornHRD, VarDict, qSNP, MuSE, Platypus, and CaVEMan.	Variant calling for single-nucleotide variants (SNVs) and short insertions/deletions (indels)	
Pindel, DELLY, Meerkat, and LUMPY	Detection of structural variants	
CNVnator, CNV-Seq, CoNIFER, ExomeCNV, Cnvkit, EXCAVATOR, HMZDelFinder, CLAMMS, WISExome, saasCNV, GSA, QDNAseq, and NxClinical	Detection of copy number variants (CNVs)	
COSMIC, dbSNP, gnomAD ClinVar VEP ANNOVAR, SnpEff, and Funcotator.	Annotation of variants (SNVs, indels, CNVs)	
ContEst, ART-DeCO, and Conpair	To assess cross-individual contamination by estimating the probability of contamination based on the allele fraction of homozygous polymorphisms	
DeTiN	To avoid erroneous filtering of true SNVs	
High-performance computing cluster, consisting of 5 nodes running the SLURM workload manager	Accelerates analyses by distributing jobs across nodes and ensuring reproducibility by storing sequencing data, genome references, aligner indexes, annotations, genomic databases, and analysis tools in a central location	
VCFtools NGS-pipe, VariantTools, vcfr, myVCF, SMuRF, Cake, and NeoMutate	Integrated tools filter the false-positive hits and provide a platform for customized variant calling pipelines for research objectives	
Mutalisk and SigMA	Mutational signatures	
MutSig2CV, dNdScv, and MutPanning	Identification of cancer driver mutations	
BRACAnalysisCDx	Detection of germline mutations of the BRCA genes to identify homologous recombination deficiency (HRD)	
HRDetect	Identifies the presence of homologous recombination repair mechanism mutations	
GATK-Mutect2, which is based on MuTect and GATK-HaplotypeCaller	To determine the tumor mutational burden (TMB)	
MANTIS, MSIseq, MSISensor, Msings, and MOSAIC	Microsatellite instability (MSI)	
CloneFinder, MACHINA, Treeomics, and LICHeE	Tumor heterogeneity	
Galaxy	Open-source web platform with several analysis tools	
LOGpc, GENT2, PROGgeneV2, SurvExpress, PRECOG, and Oncomine.	Web servers based on mRNA data for survival analyses	[85]
cBioPortal and MethSurv	Web servers based on DNA data for prognosis analyses	
TRGAted and TCPAv3.0	Web servers based on protein data for survival analyses	
Catalogue of Somatic Mutations in Cancer (COSMIC), Genomics of Drug Sensitivity in Cancer, The Cancer Genome Atlas (TCGA) data portal, DNA-Mutation Inventory to Refine and Enhance Cancer Treatment (DIRECT), My Cancer Genome Atlas Genetics Oncology, and cBio Cancer Genomics Portal	Cancer-specific databases for clinical interpretation of tumor variants	[90]
